# Thyroid Imaging Reporting and Data System for Detecting Diffuse Thyroid Disease on Ultrasonography: A Single-Center Study

**DOI:** 10.3389/fendo.2019.00776

**Published:** 2019-11-08

**Authors:** Hye Jin Baek, Dong Wook Kim, Kyeong Hwa Ryu, Gi Won Shin, Jin Young Park, Yoo Jin Lee, Hye Jung Choo, Ha Kyoung Park, Tae Kwun Ha, Do Hun Kim, Soo Jin Jung, Ji Sun Park, Sung Ho Moon, Ki Jung Ahn

**Affiliations:** ^1^Department of Radiology, Gyeongsang National University Changwon Hospital, Gyeongsang National University School of Medicine, Changwon, South Korea; ^2^Department of Radiology, Busan Paik Hospital, Inje University College of Medicine, Busan, South Korea; ^3^Department of General Surgery, Busan Paik Hospital, Inje University College of Medicine, Busan, South Korea; ^4^Department of Otorhinolaryngology-Head and Neck Surgery, Busan Paik Hospital, Inje University College of Medicine, Busan, South Korea; ^5^Department of Pathology, Busan Paik Hospital, Inje University College of Medicine, Busan, South Korea; ^6^Department of Nuclear Medicine, Busan Paik Hospital, Inje University College of Medicine, Busan, South Korea; ^7^Department of Anesthesiology and Pain Medicine, Busan Paik Hospital, Inje University College of Medicine, Busan, South Korea; ^8^Department of Radiation Oncology, Busan Paik Hospital, Inje University College of Medicine, Busan, South Korea

**Keywords:** thyroid, diffuse thyroid disease, autoimmune thyroiditis, ultrasonography, TIRADS

## Abstract

**Objective:** This study aimed to compare the ultrasonography (US) features of diffuse thyroid disease (DTD) and normal thyroid parenchyma (NTP), and to propose a structured imaging reporting system for detecting DTD.

**Methods:** This retrospective study assessed the findings for 270 consecutive patients who underwent thyroid US before thyroid surgery. The following US data were analyzed: DTD-specific features, parenchymal echotexture and echogenicity, anteroposterior diameter, glandular margin, and parenchymal vascularity. Univariate and multivariate analyses with generalized estimating equations were performed to investigate the relationship between US features and DTD. The fitted probability of DTD was analyzed by using a regression equation.

**Results:** Of the 270 patients, there were NTP (*n* = 193), Hashimoto thyroiditis (*n* = 24), non-Hashimoto lymphocytic thyroiditis (*n* = 51), Graves' disease (*n* = 1), and diffuse hyperplasia (*n* = 1). The following US features were significantly associated with DTD: decreased or increased parenchymal echogenicity, coarse parenchymal echotexture, increased anteroposterior diameter, lobulated glandular margin, and increased parenchymal vascularity. Of these, coarse parenchymal echotexture was the most significant independent predictor of DTD. The numbers of abnormal US features were positively correlated with the fitted probability and risk of DTD. The diagnostic indices were highest when the chosen cut-off criterion was category III with the largest Az value (0.867, 95% confidence interval: 0.820–0.905), yielding a sensitivity of 68.8%, specificity of 92.2%, positive predictive value of 77.9%, negative predictive value of 88.1%, and accuracy of 85.6% (*p* < 0.001).

**Conclusions:** Our sonographic reporting and data system may be useful for detecting DTD.

## Introduction

Thyroid ultrasonography (US) is commonly used to detect and characterize thyroid disease owing to its cost-effectiveness and the absence of radiation hazards (1–9). The diagnostic role of US in the management of nodular thyroid disease has been well-established (1–3); in contrast, US has a limited role in the diagnosis of diffuse thyroid disease (DTD) because clinical and laboratory findings play a more significant role in DTD. However, clinical evaluations may miss asymptomatic or subclinical DTD, while routine laboratory surveillance may be excessive in other cases (2, 10, 11). Previous studies have revealed several DTD-specific US features including micronodulation (Hashimoto thyroiditis or chronic lymphocytic thyroiditis), multifocally ill-defined hypoechoic lesions with/without tenderness (subacute thyroiditis), and thyroid inferno (Graves' disease) (6–13). Additionally, US features suggestive of DTD have been documented, including increased or decreased parenchymal echogenicity, coarse echotexture, increased or decreased anteroposterior diameter (APD) of the thyroid gland, the presence of marginal abnormality, and increased or decreased parenchymal vascularity (4–10, 14, 15). Nevertheless, the routine application of US has limited utility since it depends on the operator's experience and subjective impression (3, 15), which is important given that appropriate US diagnosis is critical when communicating the sonographic information to the physician who then determines the patient's management. To overcome potential discrepancies and miscommunications encountered in daily clinical practice, a standardized reporting system for US examinations is necessary.

Recently, a thyroid imaging reporting and data system (TIRADS) with sonographic risk stratification was developed for evaluating thyroid nodules (16–20). Additionally, structured reporting systems based on imaging modalities have also been explored for other organs such as the liver, ovary, prostate, and lung. However, to the best of our knowledge, a structured US reporting system for evaluating DTD does not yet exist. Therefore, we performed this study to compare the US features of DTD and normal thyroid parenchyma (NTP) in order to categorize US features and to propose a practical structured imaging reporting system for detecting DTD by using US. We refer to our system as the DTD-TIRADS.

## Methods

### Patients

This study was approved by Busan Paik Hospital institutional review board (IRB 18-0102). Given the retrospective nature of the investigation and the use of anonymized patient data, the requirement for written informed consent was waived. Between January and December 2015, 275 patients (228 women and 47 men with a mean age of 46.2 ± 10.7 years; range, 20–73 years) underwent thyroid US before thyroid surgery; all patients underwent thyroid surgery. After 5 patients were excluded because of poor US image quality, 270 patients (224 women and 46 men with a mean age of 46.2 ± 10.7 years; range, 20–73 years) were ultimately investigated.

### Preoperative Thyroid US

All patients underwent preoperative thyroid US, which was performed by 2 radiologists with 4 and 13 years of experience in performing thyroid US examination. High-resolution ultrasound scanners (iU 22, Phillips Medical Systems, Bothell, WA, USA; and Aplio 400, Toshiba Medical Systems, Tokyo, Japan), with 5–12 MHz and 8–15 MHz linear probes, respectively, were used. One of the 2 US instruments was arbitrarily used for each patient.

### Image Analysis and TIRADS for Diagnosing DTD

**Figure 1:**
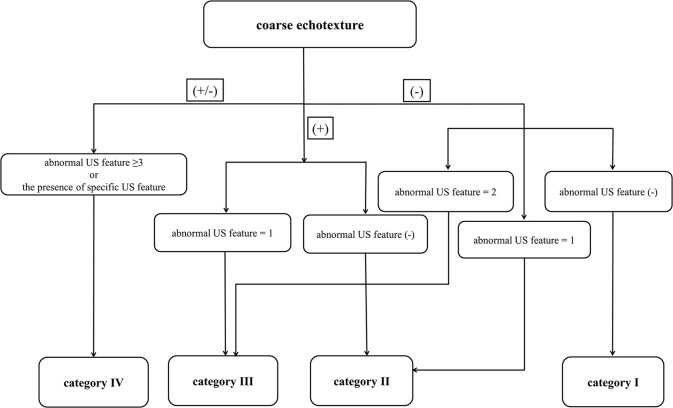
Thyroid imaging reporting and data system (TIRADS) algorithm for diffuse thyroid disease (DTD) with risk stratification based on the number of abnormal ultrasonography (US) features of DTD and the presence of DTD-specific US features. In this algorithm, the specific US features include micronodulation, multifocally ill-defined hypoechoic lesions, and thyroid inferno.

In March 2018, a single radiologist retrospectively investigated all US features of the thyroid parenchyma by using a picture archiving and communication system while blinded to clinico-serological information (such as patient age and sex, surgical outcomes, and thyroid test results) and medication history. The radiologist analyzed all US features and classified the samples. The following US features were investigated: echogenicity (normal, decreased, or increased; the strap muscle and adjacent fat tissue were utilized as the reference for determining parenchymal echogenicity); echotexture (fine [normal] or coarse); APD of the thyroid gland (normal [1–2 cm], increased [>2 cm], or decreased [<1 cm]; APDs of both lobes of the main thyroid were measured and averaged); glandular margin (smooth [normal] or lobulated); and vascularity (normal, decreased, or increased) (4, 5). Moreover, known specific US features including micronodulation (representing Hashimoto thyroiditis or chronic lymphocytic thyroiditis), multifocally ill-defined hypoechoic lesions with/without tenderness (representing subacute thyroiditis), and thyroid inferno (representing Graves' disease) were considered when determining the US category (6–9). Based on previous studies (4, 5, 14, 15), we devised a specific DTD-TIRADS algorithm (Figure 1). The US category was determined using the algorithm, which considered the number of abnormal US features as well as the presence of specific US features.

### Histopathology

A single board-certified pathologist blinded to the serological and US results retrospectively investigated the histopathological findings for the thyroid gland. Hashimoto thyroiditis was defined as progressive loss of thyroid follicular cells with replacement by lymphocytes and formation of germinal centers associated with fibrosis. Non-Hashimoto lymphocytic thyroiditis was defined as diffuse infiltration of the thyroid gland with lymphocytes and other inflammatory cells without the typical histopathological features of Hashimoto thyroiditis (such as oxyphilic metaplasia, follicular atrophy, and follicular disruption). Diffuse hyperplasia was defined as diffuse hypertrophy and hyperplasia of follicular cells with retention of the lobular architecture and no definite nodule formation. Among the cases showing diffuse hyperplasia, Graves' disease was determined based on serological results. The thyroid gland was considered to show NTP when there was no visual evidence of coexisting DTD.

### Statistical Analysis

Data were tested for a normal distribution using a Shapiro-Wilk test. We used the independent *t*-tests for continuous variables and Pearson's χ^2^ test or (for small cell values) Fisher's exact test for categorical variables when comparing the differences in US features and categories between DTD and NTP. The only continuous variable was patient age, and it is expressed as means ± standard deviations.

Associations between US features and DTD were also evaluated by using logistic regression analysis. After adjustment for all variables, multivariate logistic regression analysis with generalized estimating equations was performed to identify the US features that are significant independent predictors of DTD. The results of this analysis are presented as odds ratio (OR) estimates with corresponding 95% confidence intervals (CIs). After the analysis, we obtained a regression equation for fitting the probability of DTD. The scores and the beta coefficients obtained for each factor found to be significant on multivariate logistic regression analysis were multiplied. To evaluate the distribution of fitted probabilities associated with the number of abnormal US features, we estimated the logit (the intercept plus the sum of the beta values multiplied by the given level of each feature variable), which was subsequently used for estimating the fitted probabilities. The Cochran-Armitage trend test was used to evaluate the linear association between the number of abnormal US features and the probability of DTD. Associations between US categories and DTD were also evaluated by using logistic regression analysis. Receiver operating characteristic (ROC) curve analysis was applied to evaluate the diagnostic accuracy of DTD-TIRADS for detecting DTD. A cut-off value for the US category was determined by maximizing the sum of the sensitivity and specificity.

All statistical analyses were performed with SAS statistical software version 9.3 (SAS Institute, Cary, NC, USA). Two-sided *P* < 0.05 were considered indicative of a significant difference.

## Results

All 270 patients underwent thyroid surgery, including total thyroidectomy (155, 57.4%), hemithyroidectomy (109, 40.4%), subtotal thyroidectomy (5, 1.9%), or isthmusectomy (1, 0.4%). Histopathologic reviews following thyroid surgery revealed papillary thyroid carcinoma in 242 patients (89.6%), follicular thyroid carcinoma in 4 patients (1.5%), follicular adenoma in 11 patients (4.1%) and nodular hyperplasia in 13 patients (4.8%). The histopathological findings of the thyroid parenchyma in the 270 patients were as follows: NTP (193, 71.5%), Hashimoto thyroiditis (24, 8.9%), non-Hashimoto lymphocytic thyroiditis (51, 18.9%), Graves' disease (1, 0.4%), and diffuse hyperplasia (1, 0.4%). Of the 270 patients, 77 were subsequently classified as having DTD (mean age, 46 ± 11.3 years; range, 25–73 years; male: female ratio = 7:70) and 193 as having NTP (mean age, 46.2 ± 10.5 years; range, 20–73 years; male: female ratio = 39:154). Both DTD and NTP were significantly more common in women than in men (*p* = 0.031), but there was no significant difference in age between the 2 groups (*p* = 0.863).

**Figure 2:**
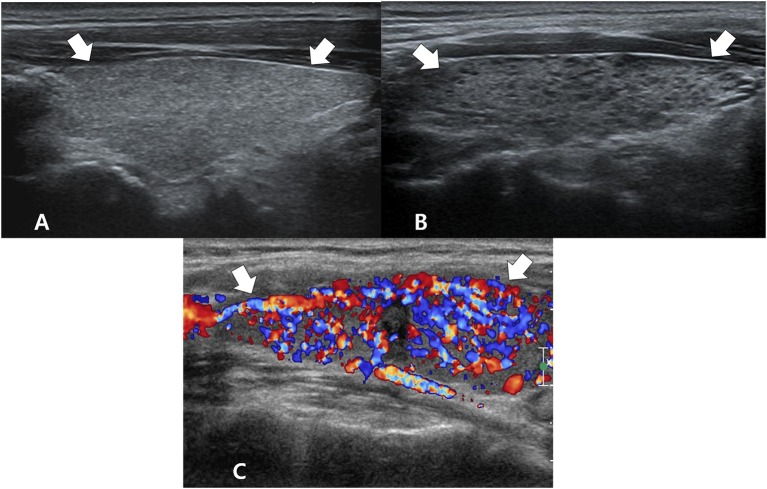
Ultrasonography images of essential and specific features for diffuse thyroid disease: coarse echotexture **(A)** and micronodulation **(B)** on longitudinal gray-scale sonograms and thyroid inferno (with poor delineation of thyroid parenchyma due to diffuse color signals) **(C)** on a longitudinal color Doppler sonogram. The arrows mark the thyroid gland.

**Figure 3:**
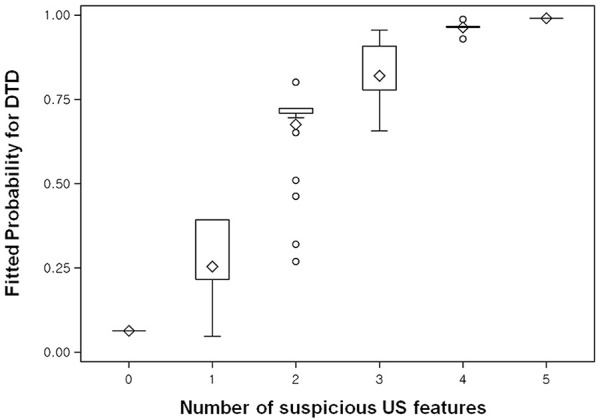
Box plot shows the fitted probabilities of diffuse thyroid disease (DTD) calculated with logistic regression by using the ultrasonography (US) features of thyroid parenchyma in the 270 patients. Probabilities are shown according to number of abnormal US features (x-axis). The lower and upper ends of the vertical lines are the minimum and maximum values of the probability of DTD, respectively. The upper horizontal line of each box represents the 75th percentile of the data set, while the lower horizontal line represents the 25th percentile. The points in the box indicated by ♢ are mean values. Lower and upper horizontal lines outside the boxes represent the 10 and 90th percentiles of the dataset, respectively. Circles represent outliers.

On univariate analysis, the following US features showed a significant association with DTD: decreased or increased parenchymal echogenicity, coarse parenchymal echotexture, increased APD, lobulated glandular margin, and decreased or increased parenchymal vascularity (Table 1). On multivariate analysis, the following US features showed a significant and independent association with DTD: decreased or increased parenchymal echogenicity, coarse parenchymal echotexture, increased APD, lobulated glandular margin, and increased parenchymal vascularity (Table 1). Of the 5 abnormal US features, coarse parenchymal echotexture was the most significant independent predictor of DTD (Figure 2). Multivariate analysis also showed that the risk of DTD increased concomitantly with the number of abnormal US features. The values of fitted probabilities were 0.064 with no abnormal US feature, 0.216–0.393 with one abnormal US feature, 0.675–0.724 with 2 abnormal US features, 0.778–0.908 with 3 abnormal US features, 0.961–0.967 with 4 abnormal US features, and 0.991 with all abnormal US features (Figure 3). The Cochran-Armitage trend test showed that the probability of DTD increased as the number of abnormal US features rose (*p* < 0.001).

**Table 1:** Association between various ultrasonography features and diffuse thyroid disease.

	No. of NTP (*n* = 193)^*^	No. of DTD (*n* = 77)^*^	**Univariate analysis**		**Multivariate analysis**	
**US features**			**Odds Ratio^†^**	**P value^‡^**	**Odds Ratio^†^**	**P value^§^**
**Echogenicity**				<0.001		
normal (*n* = 241)	186 (77.2)	55 (22.8)	1		1	
decreased (*n* = 23)	6 (26.1)	17 (73.9)	9.58 (3.6, 25.48)		1.31 (1.06, 4.91)	0.047
increased (*n* = 6)	1 (16.7)	5 (83.3)	16.91 (1.93, 147.8)		2.94 (1.37, 26.45)	0.029
**Echotexture**				<0.001		
fine (*n* = 188)	169 (89.9)	19 (10.1)	1		1	
coarse (*n* = 82)	24 (29.3)	58 (70.7)	21.49 (10.98, 42.08)		9.57 (4.42, 20.48)	<0.0001
**AP diameter**				<0.001		
normal (*n* = 252)	188 (74.6)	64 (25.4)	1		1	
decreased (*n* = 5)	4 (80)	1 (18)	0.73 (0.08, 6.69)		0.73 (0.12, 10.17)	0.346
increased (*n* = 13)	1 (7.7)	12 (92.3)	35.25 (4.49, 276.47)		8.66 (2.24, 32.89)	0.002
**Margin**				<0.001		
smooth (*n* = 252)	189 (75)	63 (21)	1		1	
lobulated (*n* = 18)	4 (22.2)	14 (77.8)	10.5 (3.33, 33.07)		3.82 (2.71, 17.26)	0.036
**Vascularity**				<0.001		
normal (*n* = 175)	152 (86.9)	23 (13.1)	1		1	
decreased (*n* = 3)	1 (33.3)	2 (66.7)	13.22 (1.15, 151.67)		6.23 (0.47, 97.82)	0.419
increased (*n* = 92)	40 (43.5)	52 (56.5)	8.59 (4.71, 15.68)		4.15 (2.69, 8.43)	0.003

* *Numbers in parentheses are percentage of each item*;

† *Numbers in parentheses are 95% confidence intervals*;

‡ *Determined with the Pearson's χ^2^ test*;

§ *Determined with logistic regression analysis. US, ultrasonography; AP, anteroposterior; DTD, diffuse thyroid disease; NTP, normal thyroid parenchyma*.

Among the 3 specific US features of DTD, micronodulation was significantly more common in DTD (6/7, 85.7%) than in NTP (1/7, 14.3%; *p* = 0.003) (Figure 2). Micronodulation was also significantly associated with DTD in both univariate and multivariate analyses (OR = 16.225; 95% CI, 1.920–89.141; *p* = 0.011 and OR = 13.714; 95% CI, 1.575–91.443; *p* = 0.028, respectively). However, there were only 2 patients with thyroid inferno, and no statistically significant relationship with DTD was observed (*p* = 0.081) (Figure 2). Moreover, there were no patients with multifocally ill-defined hypoechoic lesions.

The DTD-TIRADS US categories and histopathological results observed in the thyroid parenchyma are summarized in Table 2. The frequencies of DTD among the 270 patients as divided according to our classification system were as follows: category I (9/138, 6.5%), category II (15/64, 23.4%), category III (25/37, 67.6%), and category IV (29/31, 93.5%); all differences were significant (*p* < 0.001). The trend tests revealed that the frequency of DTD increased in the higher DTD-TIRADS US categories. Moreover, the US category was significantly associated with DTD on both univariate and multivariate analyses. Among the 31 patients classified as category IV, the following abnormal US features were observed: decreased or increased parenchymal echogenicity (30, 96.8%), coarse parenchymal echotexture (31, 100%), increased APD (10, 32.3%), lobulated glandular margin (10, 32.3%), and increased parenchymal vascularity (30, 96.8%). Among category IV patients, 2 (6.5%) were histopathologically proven to have NTP and exhibited the following 3 abnormal US features: decreased parenchymal echogenicity, coarse parenchymal echotexture, and increased parenchymal vascularity. Among the 29 patients with DTD classified as category IV, 8 (27.6%) had specific US features including micronodulation (6, 75%) and thyroid inferno (2, 25%). These 8 patients also showed at least one of the following abnormal US features: decreased parenchymal echogenicity (3, 37.5%), coarse parenchymal echotexture (6, 75%), increased APD (1, 12.5%), lobulated glandular margin (2, 25%), and increased parenchymal vascularity (6, 75%).

**Table 2:** Ultrasonography categories in the thyroid imaging reporting and data system for diffuse thyroid disease and the corresponding histopathological results for the thyroid parenchyma.

**US category**	**Histopathology**		**Univariate analysis**		**multivariate analysis**	
	NTP (*n* = 193)^*^	DTD (*n* = 77)^*^	**Odd ratio^†^**	***P*-value^‡^**	**Odd ratio^†^**	***P*-value^§^**
I (*n* = 138)	129 (93.5)	9 (6.5)	1	<0.001	1	
II (*n* = 64)	49 (76.6)	15 (23.4)	4.39 (1.89, 10.68)		3.03 (1.12, 8.19)	0.029
III (*n* = 37)	13 (35.1)	24 (64.9)	26.46 (10.18, 68.77)		10.53 (2.59, 42.76)	0.001
IV (*n* = 31)	2 (6.5)	29 (93.5)	207.833 (42.63, 1013.3)		93.32 (12.15, 716.65)	<0.0001

* *Numbers in parentheses are the percentage of each item*;

† *Numbers in parentheses are 95% confidence intervals*;

‡ *Determined with the Pearson's χ2 test*;

§ *Determined with logistic regression analysis. US, ultrasonography; NTP, normal thyroid parenchyma; DTD, diffuse thyroid disease*.

**Figure 4:**
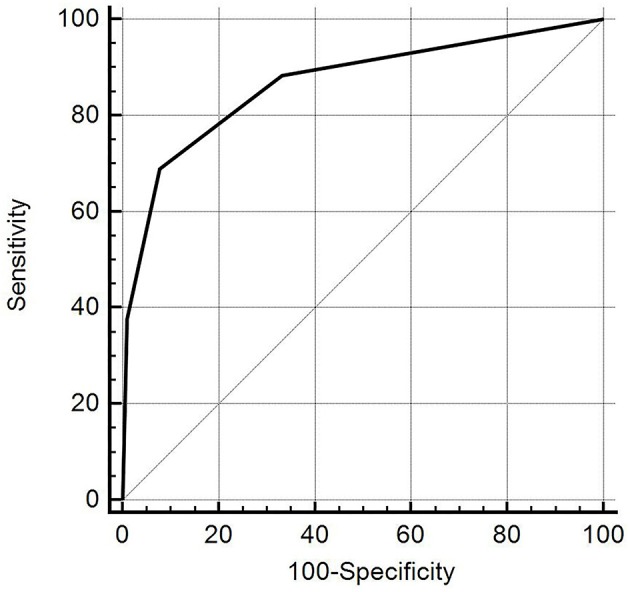
The diagnostic accuracy of the diffuse thyroid disease (DTD)-thyroid imaging reporting and data system (TIRADS) for detecting DTD in 270 patients (when the selected cut-off criterion was category III). The diagonal line represents 50% of the area under the receiver operating characteristic curve and refers to a hypothetical marker that has no discriminatory power for differentiating DTD from normal thyroid parenchyma.

ROC curve analysis showed that the DTD-TIRADS had the highest diagnostic performance when the cut-off was category III (Az = 0.867, 95% CI: 0.820–0.905), with a sensitivity of 68.8%, specificity of 92.2%, positive predictive value of 77.9%, negative predictive value of 88.1%, and accuracy of 85.6% (Figure 4).

## Discussion

We performed this pilot study to evaluate our proposed DTD-TIRADS, which is the first structured reporting system used to perform early diagnosis of DTD and facilitate its management. Early detection of asymptomatic or subclinical DTD is important in clinical practice, especially since as an association between DTD and thyroid malignancy has previously been suggested (2, 12, 13, 21–23). In the present study, we used a 4-point categorization system for DTD-TIRADS. Our classification system was based on both the fitted probability and the risk of DTD according to the number of abnormal features on US examination. Hence, DTD-TIRADS can serve as a practical and convenient reporting system in daily clinical practice.

Previous studies have attempted to validate the diagnostic performance of US for detecting asymptomatic DTD despite the controversy over the role of imaging in the diagnosis of DTD (4–10, 14, 15). The reported performances of US for DTD diagnosis have been variable, with sensitivities, specificities, positive predictive values, negative predictive values, and accuracies reported as 80.5–87.7%, 85.7–92.1%, 70.4–75%, and 81.5–97.2%, respectively. We investigated 5 US features described in previous studies (4, 5, 14, 15), and several US features including decreased or increased parenchymal echogenicity, coarse parenchymal echotexture, increased APD, lobulated glandular margin, and increased parenchymal vascularity were found to be independent predictors of DTD. Additionally, the previous studies suggested that certain US features such as micronodulation, multifocally ill-defined hypoechoic lesions with/without tenderness, and thyroid inferno were DTD-specific (6–11, 24, 25). However, our results showed that only micronodulation was significantly associated with DTD; this may be owing to the fact that a small number of patients with thyroid inferno and none with multifocally ill-defined hypoechoic lesions were included in our study. Further studies are required to clarify this aspect.

Coarse parenchymal echotexture was the most significant independent predictor of DTD among the US features examined in our study. This result supports the present algorithm that the coarse parenchymal echotexture is an essential US feature; all 31 patients classified as category IV exhibited a coarse parenchymal echotexture in this study. We also found that as the number of abnormal US features increased, the fitted probability and risk of DTD increased. The diagnostic performance of DTD-TIRADS was optimal when category III was applied as the cut-off. The results of our study suggest 2 main conclusions: First, patients with no abnormal US features and with a fitted probability of 0.064 for DTD are highly likely to have NTP. Second, patients classified as DTD-TIRADS 2, 3, or 4 (i.e., at least 1 abnormal US feature) with a fitted probability of DTD >0.216 may be candidates for thyroid function tests or other serological examinations to diagnose DTD. As such, our DTD-TIRADS classification system may be helpful.

However, several limitations of our study should be considered. First, there was unavoidable selection bias because all patients had undergone thyroid surgery. Because our institution is a referral medical center, the possibility of a higher proportion of DTD among the enrolled patients than in the general population can be expected. Second, our results were obtained by a single radiologist who reviewed the images retrospectively. Therefore, there was no cross-observer verification for the DTD imaging features. Third, all the patients underwent thyroid surgery; although this was necessary for correlating the US features with histopathological results as a reference standard, sampling bias might have occurred. Fourth, our results were obtained from a single institution with a small study population. Most of the structured imaging reporting systems for other organs were established by following consensus committee deliberations, and our classification system lacked wide clinical application in this respect. Fifth, the fitted probability of DTD for each abnormal US feature had a relatively wide range. Lastly, we did not evaluate the cost-effectiveness or follow-up management in terms of the degree of suspicion of DTD; further studies are required to address these issues.

In conclusion, we propose the DTD-TIRADS, which is based on risk stratification according to the number of abnormal US features and the presence of DTD-specific US features. In clinical practice, this DTD-TIRADS may be helpful for detecting asymptomatic or incidental DTD. Additionally, it can facilitate decision-making and can improve communication among radiologists, physicians, and patients. We expect that further studies with larger sample sizes and multiple participating institutions will validate the DTD-TIRADS proposed herein.

## Data Availability Statement

The raw data supporting the conclusions of this manuscript will be made available by the authors, without undue reservation, to any qualified researcher.

## Ethics Statement

This study was approved by Busan Paik Hospital institutional review board (IRB 18-0102). Given the retrospective nature of the investigation and the use of anonymized patient data, the requirement for written informed consent was waived. All procedures performed in this study involving human participants were in accordance with the ethical standards of the institutional and/or national research committee and with the 1964 Helsinki declaration and its later amendments or comparable ethical standards.

## Author Contributions

DHK: concept and design. HJB and DWK: acquisition of data and analysis and interpretation of data. All authors contributed to Literature review and refinement of manuscript. HJC: manuscript writing. DHK: review of final manuscript.

### Conflict of Interest

The authors declare that the research was conducted in the absence of any commercial or financial relationships that could be construed as a potential conflict of interest.
